# Menstrual Cycle Effects on Exercise-Induced Fatigability

**DOI:** 10.3389/fphys.2020.00517

**Published:** 2020-06-26

**Authors:** Hugo M. Pereira, Rebecca D. Larson, Debra A. Bemben

**Affiliations:** Department of Health and Exercise Science, The University of Oklahoma, Norman, OK, United States

**Keywords:** endurance, strength, time to task failure, progesterone and estradiol, menstrual cycle, fatigue

## Abstract

Estrogen and progesterone have distinct concentrations across the menstrual cycle, each one promoting several physiological alterations other than preparing the uterus for pregnancy. Whether these physiological alterations can influence motor output during a fatiguing contraction is the goal of this review, with an emphasis on the obtained effect sizes. Studies on this topic frequently attempt to report if there is a statistically significant difference in fatigability between the follicular and luteal phases of the menstrual cycle. Although the significant difference (the *P*-value) can inform the probability of the event, it does not indicate the magnitude of it. We also investigated whether the type of task performed (e.g., isometric vs. dynamic) can further influence the magnitude by which exercise-induced fatigue changes with fluctuations in the concentration of ovarian hormones. We retrieved experimental studies in eumenorrheic women published between 1975 and 2019. The initial search yielded 921 studies, and after manual refinement, 46 experimental studies that reported metrics of motor output in both the follicular and luteal phases of the menstrual cycle were included. From these retrieved studies, 15 showed a statistical difference between the luteal and follicular phases (seven showing less fatigability during the luteal phase and eight during the follicular phase). The effect size was not consistent across studies and with a large range (-6.77; 1.61, favoring the luteal and follicular phase, respectively). The inconsistencies across studies may be a consequence of the differences in the limb used during the fatiguing contraction (upper vs. lower extremity), the type of contraction (isometric vs. dynamic), the muscle mass engaged (single limb vs. full body), and the techniques used to define the menstrual cycle phase (e.g., serum concentration vs. reported day of menses). Further studies are required to determine the effects of a regular menstrual cycle phase on the exercise-induced fatigability.

## Introduction

Regular fluctuations in ovarian hormone levels, particularly estrogen and progesterone during the normal ovulatory cycle, produce profound alterations on the body homeostasis of women between the ages of ∼13–50 years (Marsh and Jenkins, 2002; Janse de Jonge, 2003). For example, estradiol, which is a potent estrogen, is known to strongly modulate vascular flow (Tostes et al., 2003; Joyner et al., 2015) and glycogen utilization (Hackney, 1999), whereas progesterone can increase ventilation and body temperature at rest (Marsh and Jenkins, 2002). These hormone-induced physiological alterations have the potential to produce considerable differences in performance during fatiguing exercises. Serum concentrations of estrogen and progesterone fluctuate markedly throughout the menstrual cycle, which lasts ∼23–32 days, and these fluctuations also vary among women (Stricker et al., 2006). On average ovulation occurs at day 14, and it is preceded by a follicular phase and followed by a luteal phase (on average, 12–14 days each). The hallmark of the early follicular phase (days 1–7) is the low levels of estrogen and progesterone. During the mid-follicular phase (days 7–10), estrogen slowly starts to increase and peaks in the late follicular phase (days 10–14) followed by a sharp drop just before ovulation. After ovulation, estrogen and progesterone increase during the luteal phase reaching a plateau during the mid-luteal phase (days 20–26) and later decrease again during the late luteal phase.

Fatigability is typically defined as an exercise-induced reduction in force (Enoka and Duchateau, 2008), and this construct may be influenced by the individuals’ subjective perceptions during the task (Kluger et al., 2013). Fatigability is also task dependent (Enoka and Stuart, 1992; Bigland-Ritchie et al., 1995). More specifically, the demands of the task (e.g., isometric vs. dynamic contractions) will stress different physiological sites that in turn also receive strong regulatory input from the ovarian hormones. Any influence these hormones have during fatiguing contractions across a menstrual cycle is complex because of the several systems involved (cardiorespiratory, neuromuscular, etc.). Several studies attempted to address this topic by investigating the effects of the menstrual cycle on the exercise-induced reduction in force (see results). Studies evaluating the effects of menstrual cycle on the fatigability typically report if there is a statistical significant difference between cycle phases (e.g., follicular vs. luteal), and while a significant difference (*P*-value) can inform the reader about the probability of the event, it does not indicate its magnitude (Cohen, 1988). Effect sizes are a useful tool to quantify the magnitude of difference between conditions, such as the magnitude of fatigability across the menstrual cycle. Understanding the effect size of the fluctuations in the ovarian hormones together with the *P*-value has important implications. For example, one can determine if an intervention has a greater effect size than the regular fluctuations promoted by the concentrations of estrogen and progesterone, given that both have a significant effect. Additionally, the obtained effect sizes may provide valuable information for studies that need to control and test for fatigability across the cycle.

The goal of this mini-review is to summarize the effects of the ovarian hormonal fluctuations on the exercise-induced reduction in force during fatiguing contractions with emphasis on the effect size. Our hypothesis is that the menstrual cycle phase will influence the exercise-induced decline in force, but the effects will vary according to the task performed and the limb used. Data was retrieved from database searches using a combination of terms: “menstrual cycle,” “menstrual phase,” “menstruation,” “progesterone,” “estrogen,” “follicular phase,” “luteal phase,” “fatigue,” “fatigability,” “time to task failure,” “endurance performance.” Moreover, wildcard terms such as “menstru”^∗^ and “fatig”^∗^ were also used. In this initial search, 921 studies were obtained. The retrieved manuscripts were further refined by including experimental studies in eumenorrheic women not taking oral contraceptives published in English between 1975 and 2019. We focused on studies that describe the metrics of motor output (time to failure, maximal voluntary contraction, power, work, etc.), and we included studies that reported exercise-induced reduction in force during both the luteal and follicular phases. These inclusion criteria returned 46 experimental studies used in this review.

### Data Analyses

To estimate the effects of the menstrual cycle on the exercise-induced fatigability, we calculated the Hedges’s *g* effect size, as it is adequate for small sample sizes typically found in the retrieved studies (Hedges, 1981). The mean difference between the follicular phase and the luteal phase was calculated for each variable using the follicular phase as a reference (e.g., Hedge’s *g* = follicular phase – luteal phase/pooled standard deviation), so a positive effect size indicates that the follicular phase had greater values on average. For each study, we carefully indicated the specific menstrual cycle phase used for the effect size calculation (e.g., early follicular vs. mid follicular; reported in each table) to account for variations across studies. It was not possible to calculate the effect size for the manuscripts that did not report exact standard deviations and/or average values (for example studies that included standard deviation or averages only in the figures). Additional interpretations throughout the text were obtained by calculating the percentage difference between the cycle phases. Tables 1–6 also indicate if the original report found a statistical significant difference (*P* < 0.05) between the phases of the menstrual cycle.

**TABLE 1:** Time to task failure.

**References**	***n***	**Training status**	**BMI (kg/m^2^)**	**Age (yr)**	**Muscle**	**Contraction type**	**Intensity**	**Cycle phase**	**Effect size [95%CI]**	**Stat diff**
Ansdell et al. (2019)	30	Not reported	24	25 ± 4	KE	Isometric intermittent	60% of MVC	*e*F vs. *m*L	−0.84 [−1.37; −0.32]	Y (↑ *m*L)
Birch and Reilly (1999)	17	Not reported	21	18–32	Whole body	Isometric sustained	45% of MVC	*m*F vs. *m*L	0.24 [−0.43; 0.92]	N
Birch and Reilly (2002)	10	Not reported	–	24 ± 3	Whole body	Isometric sustained	45% of MVC	F vs. L	−0.22 [−1.10; 0.66]	N
Hoeger Bement et al. (2009)	20	Not reported	23	21 ± 1	Elb. Flex	Isometric sustained	25% of MVC	*m*F vs. *m*L	0.02 [−0.61; 0.64]	N
Jarvis et al. (2011)	11	Not endurance trained	23	33 ± 10	Handgrip	Isometric sustained	40% of MVC	*e*F vs. *m*L	–	N
Petrofsky et al. (1976)	4	Not reported	21	25 ± 5	Handgrip	Isometric sustained	40% of MVC	F vs. L	–	Y (↑ F)
Petrofsky et al. (2007)	8	Non-athletes	18	25 ± 9	Handgrip	Isometric sustained	20,40, and 60% of MVC	F vs. L	–	Y (↑ F)
Tenan et al. (2016)	9	Recreationally active	–	25 ± 5	KE	Isometric sustained	25% of MVC	*e*F vs. *l*F vs. *m*L vs. *l*L	–	N
Wirth and Lohman (1982)	10	Not reported	–	18–33	Handgrip	Isometric sustained	50% of MVC	F vs. L	−0.33 [−1.21; 0.55]	N

**eF, Early follicular; Elb. Flex, Elbow Flexors; F, Follicular; KE, Knee extensors; KF, Knee flexors; L, Luteal; lF, Late follicular; MVC, Maximal Voluntary Contraction; mL, Mid luteal; lL, Late luteal; N, No statistical difference between the phases; Y, yes, there was a statistical difference between the menstrual cycle phases. Upward arrow indicates which menstrual cycle phase had greater time to task failure (i.e., lower fatigability). yr, years old.**

**TABLE 2:** Percentage decline in maximal strength after a fatiguing contraction.

**References**	***n***	**Training status**	**BMI (kg/m^2^)**	**Age (yr)**	**Muscle**	**Contraction type**	**Intensity**	**Cycle phase**	**Effect size [95%CI]**	**Stat diff**
Ansdell et al., 2019	30	Not reported	24	25 ± 4	KE	Isometric intermittent	60% of MVC	*e*F vs. *m*L	–	N
					KE				0.10 [−0.50; 0.71]	N
Dibrezzo et al., 1988	21	Not reported	–	18–36		Isokinetic 240 °/s	Maximum	*e*F vs. L		
					KF				0.03 [−0.58; 0.63]	N
Friden et al., 2003	10	No more than two training session/week	23	25 ± 4	KE	Isokinetic 120 °/s	Maximum	F vs. L	−0.16 [−1.04; 0.72]	N
					KF	Isokinetic 240 °/s			0.19 [−0.52; 0.91]	N
Janse de Jonge et al., 2001	15	Not reported	–	30 ± 8			Maximum	*l*F vs. L		
					KE	Isokinetic 240 °/s			−0.01 [−0.72; 0.71]	N
Nicolay et al., 2008	11	Not reported		17–30	Hand	Isometric Intermittent	Maximum	*e*F vs. L	0.94 [0.06; 1.82]	Y (↑ L)
Pallavi et al., 2017	100	Not reported	21	18 ± 1	Finger Flexors	Isotonic	–	F vs. L	1.10 [−1.39; −0.80]	Y(↑ F)

**eF, Early follicular; F, Follicular; KE, Knee extensors; KF, Knee flexors; L, Luteal; lF, Late follicular; MVC, Maximal Voluntary Contraction; mL, Mid luteal; N, No statistical difference between the phases; Y, yes, there was a statistical difference between the menstrual cycle phases. Upward arrow indicates which menstrual cycle phase had greater decline in maximal strength (i.e., greater fatigue). yr, years old.**

**TABLE 3:** Maximal voluntary strength.

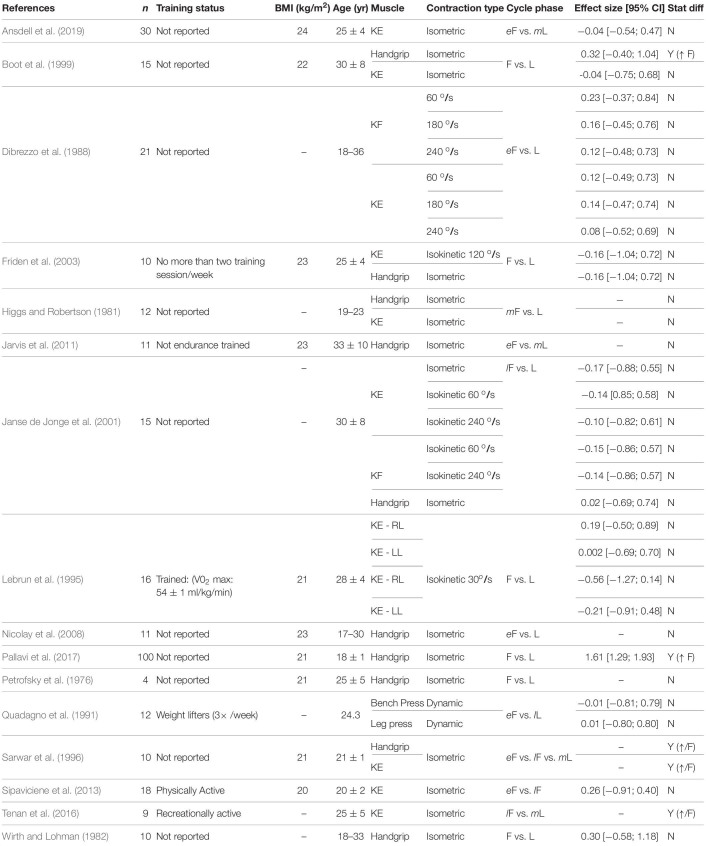

**eF, Early follicular; F, Follicular; KE, Knee extensors; KF, Knee flexors; lL, Left Leg; RL, Right leg; L, Luteal; lF, Late follicular; mL, Mid luteal; N, No statistical difference between the phases; Y, yes, there was a statistical difference between the menstrual cycle phases. Upward arrow indicates which menstrual cycle phase had greater strength. yr, year old.**

**TABLE 4:** Cycling endurance time.

**References**	***n***	**Training status**	**BMI (kg/m^2^)**	**Age (yr)**	**Protocol**	**Intensity**	**Cycle phase**	**Effect size [95%CI]**	**Stat diff**
Bailey et al. (2000)	9	Trained (VO_2_ peak: 50 ± 4 mL/kg/min)	22	27 ± 7	Constant	70% of VO_2_ peak	F vs. L	–	N
Campbell et al. (2001)	8	Endurance trained: (VO_2_ peak: 54 ± 1 mL/kg/min	21	24 ± 2	Mixed	Time trial test after cycling 2 h at 70% VO_2_ peak	F vs. L	−1.31 [−2.39; −0.23]	Y (↑ F)
Jurkowski et al. (1981)	9	VO_2_ peak: 50 ± 4 mL/kg/min	21	22 ± 1	Mixed	20 min at 1/3 max power, 20 min at 2/3 of max power and later 90% of max power until failure	F vs. L	−2.67 [−3.9; −1.4]	Y (↑ L)
Janse et al. (2012)	8	Recreationally active (VO_2_ peak: 40 ± 7 mL/kg/min)	24	24 ± 4	Mixed	60 min at 60% of VO_2_ max + incremental until failure	*e*F vs. *m*L	0.23 [−0.75; 1.22]	N
Kraemer et al. (2006)	8	Active (VO_2_ peak not reported)	22	27 ± 1	Incremental	1 kp/2 min	F vs. L	1.14 [0.09; 2.2]	N
Lara et al. (2019)	13	Well-trained (VO_2_ peak: 48 ± 7 mL/kg/min)	21	31 ± 6	Incremental	25 W/min until failure	*e*F vs. *m*L	–	N
McLay et al. (2007)	9	Moderately trained (VO_2_ Peak: 50 ± 4 mL/kg/min)	24	25 ± 7	Time trial	Self-paced	*m*F vs. *m*L	−0.14 [−1.07; 0.78]	N
Nicklas et al. (1989)	6	Moderately trained (VO_2_ Peak: 45 ± 2 mL/kg/min)	22	26 ± 2	Constant	70% of VO_2_ peak	*m*F vs. *m*L	−0.75 [−1.92; 0.42]	N
Oosthuyse et al. (2005)	11	VO_2_ Peak: 30–45 mL/kg/min	22	24 ± 3	Time trial	Self-paced	*e*F vs. *l*F vs. *m*L	0.03 [–0.81; 0.86]	N
Redman et al. (2003)	14	Sedentary (VO_2_ Peak: ∼42 mL/kg/min)	23	21 ± 4	Incremental	25 W/2 min until failure	F vs. L	0.00 [−0.74; 0.74]	N

**eF, Early follicular; F, Follicular; mF, Mid Follicular; mL, Mid luteal; L, Luteal; N, No statistical difference between the phases; Y, yes, there was a statistical difference between the menstrual cycle phases. Upward arrow indicates which menstrual cycle phase had lower fatigability. yr, years old.**

**TABLE 5:** Running endurance time.

**References**	***n***	**Training status**	**BMI (kg/m^2^)**	**Age (yr)**	**Protocol**	**Intensity**	**Cycle phase**	**Effect size [95%CI]**	**Stat diff**
Bandyopadhyay and Dalui (2012)	45	Sedentary (estimated VO_2_ max: ∼39 ml/kg/min	21	23 ± 3	Constant	8–10 Km/h	*e*F vs. L	−6.77 [−7.84; −5.70]	Y (↑ L)
Beidleman et al. (1999)	8	Physically active (VO_2_ max: ∼ 47 ml/kg/min)	22	33 ± 3	Constant	70% of VO_2_ maximum	F vs. L	−0.10 [−1.08; 0.88]	N
Bemben et al. (1995)	5	Moderately active (VO_2_ max: ∼ 43 ml/kg/min)	24	22 ± 4	Incremental	Increased 1% grade/min	*e*F vs. *m*L	0.02 [−1.22; 1.26]	N
Bryner et al. (1996)	3	VO_2_ max: ∼ 40 ml/kg/min		18–30	Constant	80% of maximum HR	*m*F vs. *m*L	0.17 [−1.43; 1.78]	N
De Souza et al. (1990)	16	Trained (VO_2_ max: 53 ± 4 ml/kg/min)	19	29 ± 4	Incremental	2% every 2 min until max	*e*F vs. *m*L	0.32 [−0.37; 1.02]	N
						90% of VO_2_ maximum	F vs. L	0.25 [−0.94; 0.45]	N
Lebrun et al. (1995)	16	Trained (VO_2_ max: 54 ± 1 ml/kg/min)	21	28 ± 4	Constant				
						8 mph at 20% incline	F vs. L	0.04 [−0.65; 0.74]	N
McCracken et al. (1994)	9	Trained, non-athletes (VO_2_ max: 46 ± 3 ml/kg/min)	-	18–32	Incremental until 30 min then constant after it	35, 60, 75% of VO_2_ max before 30 min. Then a 90% VO_2_ max was performed after 30 min	*m*F vs. *m*L	0.06 [−0.86; 0.99]	N
Higgs and Robertson (1981)	12	Not reported	-	19–23	All out, workload greater than VO_2_ max	Maximum	*e*F vs. *l*F	–	Y (↑*l*F)

**eF, Early follicular; F, Follicular; HR, Heart Rate; mF, Mid follicular; mL, Mid luteal; L, Luteal; lF, Late follicular; N, No statistical difference between the phases; Y, yes, there was a statistical difference between the menstrual cycle phases. Upward arrow indicates which menstrual cycle phase had greater endurance time (i.e., less fatigability). yr, years old.**

**TABLE 6:** Ratings of perceived exertion.

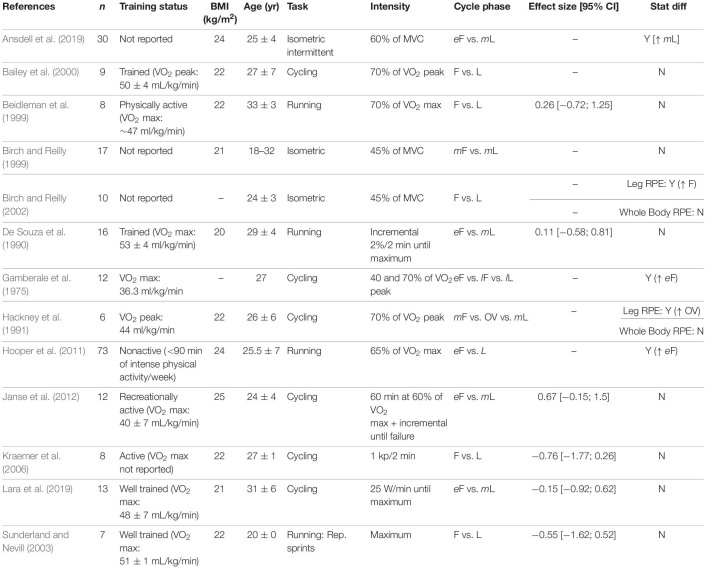

**eF, Early follicular; F, Follicular; mL, Mid luteal; L, Luteal; lF, Late Follicular; lL, Late luteal; OV, Ovulatory; N, No statistical difference between the phases; Y, yes, there was a statistical difference between the menstrual cycle phases. Upward arrow indicates which menstrual cycle phase had greater Ratings of Perceived Exertion (greater perceived fatigability). yr, years old.**

### Fatigability During Isometric, Isotonic, and Isokinetic Tasks

#### Time to Task Failure

The menstrual cycle phase has equivocal effects on the time to task failure (Table 1). The different results across studies may be a consequence of the type of muscle contraction (e.g., intermittent vs. sustained) or the muscle group used (upper vs. lower extremity vs. whole body). For example, for the knee extensor muscles, some reported a ∼26% greater time to task failure during the mid-luteal phase compared to the early follicular phase during an intermittent isometric contraction (effect size: −0.84; Table 1; Ansdell et al., 2019). During sustained isometric contractions with the knee extensors, although the follicular phase had a trend to show greater time to task failure, there were no statistical differences between the menstrual cycle phases (Tenan et al., 2016). For the upper extremity muscles, three studies showed no difference between the luteal phase and the follicular phase during a sustained isometric contraction with the hand or elbow flexor muscles (Wirth and Lohman, 1982; Hoeger Bement et al., 2009; Jarvis et al., 2011). However, two studies reported that the follicular phase had approximately a 7–60% longer time to task failure than the luteal phase during a sustained isometric contraction with the hand muscles (Petrofsky et al., 1976, 2007). The greater endurance time during the follicular phase was larger at the lower contraction intensity compared to larger ones (20 vs. 60% of maximum) (Petrofsky et al., 2007).

#### Fatigue Index

Six studies reported fatigue index calculated as the percent decline in maximal voluntary contraction relative to baseline. For the lower extremity muscles, the menstrual cycle phase did not influence the fatigue index (Dibrezzo et al., 1988; Janse de Jonge et al., 2001; Friden et al., 2003; Ansdell et al., 2019). However, for the upper extremity muscles, some indicated a ∼4% greater decline in force (i.e., greater exercise-induced fatigability) during the follicular phase compared to the luteal phase (52 ± 4 vs. 56 ± 4% of baseline, respectively) (Pallavi et al., 2017), whereas others reported a ∼15% larger reduction in force during the luteal phase (follicular: 96 ± 19 vs. luteal: 81 ± 11% baseline) (Nicolay et al., 2008; Table 2). Differences in the task performed may also have contributed to the mixed results. For example, the fatigue index was assessed in the upper extremity muscles during isotonic and isometric intermittent tasks whereas for the lower extremity the tasks were isokinetic and isometric intermittent (Table 2). Isometric intermittent was performed in both the upper extremity and lower extremity muscles, and the luteal phase had greater exercise-induced fatigability (i.e., greater decline in force) for the former muscles only.

#### Maximal Strength

We examined changes in maximal strength across the menstrual cycle as they can strongly influence the time to task failure (Carlson and McCraw, 1971; Hunter and Enoka, 2001). Out of the studies retrieved, only four found a statistical difference between the follicular phase and the luteal phase (Table 3) and better detailing of their effect sizes according to the limb involved and the task performed is described below:

##### Isometric tasks

For the lower extremity muscles, some indicated no change across the menstrual cycle (Boot et al., 1999; Janse de Jonge et al., 2001; Bambaeichi et al., 2004; Sipaviciene et al., 2013; Ansdell et al., 2019; Sung and Kim, 2019), whereas others indicated the maximum strength was 10% greater during the follicular and ovulatory phases (Sarwar et al., 1996; Tenan et al., 2016). For the upper extremity muscles, the results are also mixed as some reported 5–20% greater strength during the follicular (i.e., low levels of estrogen and progesterone) (Bassey et al., 1995; Boot et al., 1999; Pallavi et al., 2017), and 10% greater strength during the ovulatory or luteal phases (i.e., greater estrogen concentration compared to the follicular phase) (Phillips et al., 1996; Sarwar et al., 1996). Others showed no changes across the cycle (Janse de Jonge et al., 2001; Friden et al., 2003; Nicolay et al., 2008; Jarvis et al., 2011; Sakamaki-Sunaga et al., 2016; Table 3).

##### Isokinetic tasks

No effect of the menstrual cycle phase was observed during tests performed with the knee extensors and flexors at 30, 60, 90, 120, or 240 degrees/second (Dibrezzo et al., 1988; Lebrun et al., 1995; Janse de Jonge et al., 2001; Friden et al., 2003; Bambaeichi et al., 2004; Sipaviciene et al., 2013; Wikstrom-Frisen et al., 2017).

##### Dynamic constant resistance

No influence of the menstrual cycle phase was observed for one-repetition maximum (1-RM) during the bench press, bicep curl, half-squat, and leg press tests (Quadagno et al., 1991; Kraemer et al., 1995; Markofski and Braun, 2014; Sakamaki-Sunaga et al., 2016; Romero-Moraleda et al., 2019a, b).

### Cycling

Table 4 summarizes the data for endurance time during a cycling task. The follicular and luteal phases produced similar time results in a 15–30 km event (time trial) (Oosthuyse et al., 2005; McLay et al., 2007), or when cycling at 60 or 70% of the VO_2_ peak to exhaustion (Nicklas et al., 1989; Bailey et al., 2000; Janse et al., 2012). During an incremental protocol, the menstrual cycle had no effect on the endurance time (Redman et al., 2003; Kraemer et al., 2006) or the power output (Casazza et al., 2002; Redman et al., 2003; Smekal et al., 2007). Conversely, when the intensity was increased to a 90% VO_2_ peak after the individuals had cycled at lower intensities earlier in the protocol, the time to task failure was ∼50% longer during the luteal phase compared to the follicular phase (Jurkowski et al., 1981).

The influence of the menstrual cycle phase on exercise-induced fatigability during cycling was also evaluated during single or repeated sprints, and the cycle phase had no effect on the peak power or the drop-off in work during the sprints (Giacomoni et al., 2000; Middleton and Wenger, 2006; Shaharudin et al., 2011; Wiecek et al., 2016; Tounsi et al., 2018). However, the average work was slightly greater during the luteal phase compared to the follicular phase for the 10 × 6 s sprint (39.3 vs. 38.3 J.kg^–1^, respectively) (Middleton and Wenger, 2006).

### Running

The time to exhaustion was similar between the follicular and luteal phases when running at 70 or 90% of the VO_2_ max, 80% of the maximum heart rate, or performing an anaerobic speed test (i.e., 8 miles/hour at a 20% incline), or an incremental protocol (McCracken et al., 1994; Bemben et al., 1995; Lebrun et al., 1995; Bryner et al., 1996; Beidleman et al., 1999; Table 5). The menstrual cycle phase had no effect on the maximum running velocity (Burrows and Bird, 2005). However, at a supramaximal intensity (greater than VO_2_ max), women had a greater endurance time (∼15%) during the late follicular phase compared to the early follicular phase (Higgs and Robertson, 1981). Endurance time was also longer (∼17%) during the luteal and mid follicular phases compared to the early follicular phase in a constant protocol at 8–10 km.h^–1^ (Bandyopadhyay and Dalui, 2012). During repeated sprints there was no difference between the follicular and luteal phases in the distance run (Sunderland and Nevill, 2003; Julian et al., 2017), or peak power (Tsampoukos et al., 2010).

Differences in running economy across the menstrual cycle have been suggested to impact running performance (Williams and Krahenbuhl, 1997). These suggestions are based on the observation that concentrations of progesterone, that peak in the luteal phase, are positively associated with ventilation at rest (Skatrud et al., 1978) and increased inspiratory muscle endurance during a breathing test (Chen and Tang, 1989). Ventilation during exercise, however, has mixed results. While some report greater ventilation during the luteal phase (Williams and Krahenbuhl, 1997), others report no change across the cycle (De Souza et al., 1990; Lebrun et al., 1995). A confounding factor may involve the training status of the individuals. Athletes, for example, had lower changes in ventilation across the menstrual cycle compared to non-athletes during an incremental cycling test (Schoene et al., 1981). Whether any change in running economy across the menstrual cycle is translated to performance fatigability, represented by the endurance time or sprint time, is not well-understood.

### Ratings of Perceived Exertion (RPE)

The exercise-induced reduction in force or power is potentially influenced by the individual’s psychological state and perception of the task performed (Mosso et al., 1903; Enoka and Duchateau, 2016). Because progesterone concentration may be associated with perceptual responses (Gonda et al., 2008; Romans et al., 2013; Reynolds et al., 2018), we also investigated the influence of menstrual cycle phase on the individual’s perception during the task performed, which was typically estimated with the RPE scale. The effects of the menstrual cycle phase on the RPE during fatiguing tasks was mixed. The effect sizes were variable and in both directions without a common trend (i.e., to the follicular or luteal phases) (Table 6; De Souza et al., 1990; Beidleman et al., 1999; Sunderland and Nevill, 2003). For example, for running the RPE was similar between the follicular and luteal phase (1% difference between the follicular and luteal phases with no statistical difference) (De Souza et al., 1990; Beidleman et al., 1999; Sunderland and Nevill, 2003) or approximately 2 points greater during the early follicular compared to luteal phase (Hooper et al., 2011). For cycling, the majority of studies do not show a statistical difference across the menstrual cycle (Stephenson et al., 1982; Bailey et al., 2000; Kraemer et al., 2006; Janse et al., 2012; Lara et al., 2019). However, one study reported the RPE was ∼1 point greater during the early follicular phase (menstruation) compared with the late follicular and late luteal when cycling either at 40 or 70% of VO_2_ max (Gamberale et al., 1975). Conversely, the local leg RPE during cycling was 1–2 points greater during the ovulatory phase, but there was no change in total body RPE across the cycle (Hackney et al., 1991). During a functional isometric fatiguing task using the whole body (similar to a lifting a box from the ground), there was no effect of menstrual cycle phase on the total body RPE results (Birch and Reilly, 1999, 2002), but the local leg RPE was greater during the follicular compared to the luteal phase (Birch and Reilly, 2002). During an intermittent isometric fatiguing contraction with the knee extensor muscles, the RPE was greater during the luteal phase compared to the follicular phase (Ansdell et al., 2019).

## Discussion

The goal of this mini-review is to summarize the magnitude of changes in the exercise-induced reduction in force across the regular menstrual cycle. From the retrieved studies, 9 out of 32 indicated the menstrual cycle phase had an effect (i.e., statistical difference) on the exercise-induced reduction in force (i.e., lower time to task failure or greater fatigue index) during either a single limb exercise with the lower or upper extremity, or whole body exercise (constant or incremental) (Tables 1, 2, 4, 5). The results are also equivocal for maximal strength (4 out of 16 showing statistical difference, Table 3) and perception of the effort (5 out of 13 showing statistical difference, Table 6). The calculated effect size was variable, and the exercise-induced fatigability was shown to be greater either at the luteal or the follicular phase (-6.77; 1.61, respectively) (Tables 1–6). Task dependency of fatigability (i.e., exercise mode, intensity of the task, limb involved and environment) may influence the equivocal results across studies. Confounding factors such as the serum concentration of ovarian hormones, presence of ovulatory vs. anovulatory cycles, training and nutritional status should be considered in future studies. There is ample opportunity for investigations on the effects of regular hormonal fluctuations accounting for the task performed, environment and the limb involved. Below is a discussion about the potential mechanisms driving the influence of the above-mentioned factors on the performance fatigability across the menstrual cycle.

### Metabolism

Metabolic responses are largely influenced by the concentration of estrogen with potential implications for motor performance (Lebrun, 1993; Wismann and Willoughby, 2006; Oosthuyse and Bosch, 2010). For example, an animal study indicated that supplementation of estrogen in ovariectomized rats increased endurance time of the animals and this finding was associated with the muscle glycogen-sparing effect (Kendrick et al., 1987). The greater concentration of estrogen during the luteal phase has potential to reduce fatigability in humans. During a cycling task performed at 65 or 70% of VO_2_ max, women had less glycogen utilization (Hackney, 1999; Devries et al., 2006) and lower leg RPE values (Hackney et al., 1991) when the level of estrogen was high (i.e., mid-luteal phase) compared to the mid-follicular phase (low levels of estrogen). However, the upper and lower extremity muscles have different metabolic responses to ovarian hormones that may influence the exercise-induced fatigability. More specifically, the arm muscle exercise was shown to require greater reliance on glycogen compared to leg muscles (Ahlborg and Jensen-Urstad, 1991). Because the glycogen sparing is somewhat greater during the luteal phase (Wismann and Willoughby, 2006), perhaps the lesser reliance on glycogen in the legs is enhanced during the luteal phase allowing less fatigability compared to the arm muscles. This rationale would explain the greater endurance time during high levels of estrogen (i.e., luteal phase) for a knee extensors task performed with minimal impact on blood flow (Ansdell et al., 2019), whereas for the upper extremity minimal levels of estrogen paralleled a negligible (Hoeger Bement et al., 2009; Jarvis et al., 2011) or a greater (Petrofsky et al., 1976, 2007) endurance time. In this review, each table also indicates the training status of the individuals tested, as it can influence substrate availability in women (Ruby and Robergs, 1994; Carter et al., 2001). Another potential metabolism-associated factor driving the large variability in fatigability across the menstrual cycle between individuals, and perhaps explaining the lack of agreement between studies, is the estrogen-to-progesterone concentration ratio. In brief, progesterone is typically associated with increased catabolism whereas estrogen suppresses catabolism (Lamont et al., 1987; Bailey et al., 2000). Studies conducted in individuals with a lower estrogen to progesterone ratio typically fail to show differences in motor performance between the follicular and luteal phases (for a detailed review, see Oosthuyse and Bosch, 2010). For example, in presence of a larger estrogen-to-progesterone concentration ratio, cycling and running endurance times were longer (Jurkowski et al., 1981; Nicklas et al., 1989) compared with lower ratios (Beidleman et al., 1999; Bailey et al., 2000) (∼18–21 vs. 8–12 Pmol/nmol, respectively). Other factors influencing glucose availability, such as nutritional status and exercise intensity, may explain the conflicting findings in fatigability across the menstrual cycle (Lebrun, 1993; Oosthuyse and Bosch, 2010; Isacco et al., 2012), and perhaps should be considered when designing future studies addressing the menstrual cycle effects on the exercise-induced fatigability.

### Temperature

Fluctuations in the concentration of ovarian hormones may have consequences on exercise-induced fatigability because of changes in the core temperature. Progesterone acts in the hypothalamus increasing the body set point temperature (Stephenson and Kolka, 1993). Consequently resting body temperature is slightly higher (∼0.3–0.5°C) during the luteal phase compared with the follicular phase (Marshall, 1963; Nakayama et al., 1975). The greater body temperature during the luteal phase was shown to alter the perceptual and physiological responses during the exercise. For example, during a 60 min. cycling exercise, the greater core temperature paralleled the higher heart rate and ratings of perceived exertion during the luteal phase compared to the follicular phase, but only in the women who showed a large rise in serum progesterone concentration during the luteal phase (Pivarnik et al., 1992). During a sustained isometric contraction with the hand muscles, immersing the arm in warm water (37°C) decreased the time to task failure compared to the exercise performed at 24°C (Petrofsky et al., 1976, 2007). Both these results suggest the progesterone-induced increase in the body temperature could explain the increased fatigability in the luteal phase. However, other observations showed less exercise-induced fatigability at the luteal phase during isometric intermittent contractions with the lower extremity muscles, and whole body exercise and therefore do not agree with this hypothesis (Jurkowski et al., 1981; Bandyopadhyay and Dalui, 2012; Ansdell et al., 2019; Tables 1, 4, 5). Perhaps the menstrual cycle–induced alterations in metabolism in the arm and leg muscles (detailed above) have greater impact on performance fatigability than temperature.

Regulations in body temperature can also have implications for fatiguing exercises performed in hot environments. More specifically, if adequate body thermoregulation during exercise cannot account for the greater baseline temperature showed in the luteal phase, hot and humid environmental conditions may have a strong impact on the exercise-induced fatigability. Accordingly, individuals cycling in a hot environment (32°C, 60% humidity), had reduced time to exhaustion (∼6%) during the luteal phase compared to the follicular phase (Janse et al., 2012). The menstrual cycle phase, however, had no influence on the distance run during a repeated sprint test performed in a less extreme condition (31°C, 23% humidity) (Sunderland and Nevill, 2003). This latter conflicting result may be a consequence of the task performed as well as the ratio of progesterone to estrogen in the participants (Stephenson and Kolka, 1993). Although increased concentrations of progesterone can increase the core temperature, estrogen administration can attenuate these thermoregulatory effects, and a balance between the two hormones may influence the response to thermoregulation during exercise (Oosthuyse and Bosch, 2010).

### Limitations

This review has some limitations inherent to the studies retrieved. One of them is the classification of the menstrual cycle phase. Some early studies used a somewhat arbitrary criteria (e.g., ovulatory vs. pre-menstrual vs. post-menstrual) assuming fertility in a regular 28-day cycle, which may not correspond to the phases determined by modern hormonal documentation or the presence of anovulatory cycles. Because of the variability in how follicles grow within the ovaries or presence of anovulatory cycles, which results in considerable discrepancies in the production of ovarian hormones among women, steps to better determine the phase of the cycle were recently proposed. They include a three-step method that comprise the evaluation of serum concentration, cycle mapping, and urinary ovulation prediction (Schaumberg et al., 2017; Sims and Heather, 2018). Future studies using this strategy can provide valuable information regarding the influence of the estrogen to progesterone concentration ratio on the exercise-induced reduction in force. Others have investigated the inconsistent results across the studies with emphasis on the technique used to identify the phase of the menstrual cycle (i.e., serum concentration vs. other methods such as day of menses or body temperature) and found out that only 44% of the studies actually measured the concentration of the female hormones (Janse et al., 2019). The early studies may also be influenced by the self-expectancy of individuals performing an exercise during the menstruation, as myths and cultural restrictions were perhaps more evident leading to negative attitude toward menstruation (Lebrun, 1993). To account for the above-mentioned limitations, the current review chose to present the effect size, the statistical significance and the menstrual cycle phase of each study separately and not compiled in a meta-analysis. Another limitation is the small sample size and presence of type I and type II errors in the retrieved studies. For example, a typical error of 10%, independent of the cycle phase, was found across visits when measuring the knee extensors maximal strength (Ansdell et al., 2019) or running endurance time (Bryner et al., 1996). Caution should be used when menstrual cycle related changes in motor output are below the error of the measurement. Future studies should consider using a control group to determine the error in the measurement independent of the hormonal fluctuations.

## Summary

This review indicates the effects of the menstrual cycle phase on performance fatigability has mixed results. Although several studies did not indicate a difference between the classical definitions of luteal and follicular phases, some report greater fatigability during the luteal phase whereas others show greater fatigability during the follicular phase. Disagreement across studies may be a consequence of the limb (upper vs. lower) and task differences (dynamic vs. isometric), as well as inconsistencies in the definitions of the phase of the menstrual cycle and the relative concentration of progesterone to estrogen. As the number of retrieved studies was limited, there is an ample opportunity for addressing the impact of the regular menstrual cycle phase on the exercise-induced fatigability. Future studies should consider quantifying the measurement error and using a prospective design that allows carefully mapping the menstrual cycle, quantifying the estrogen to progesterone concentration ratio, and verifying the presence of the ovulatory and anovulatory cycles, as they may modify the hormonal fluctuations responsible for changes in the exercise-induced fatigability.

## Author Contributions

HP designed the study. All authors interpreted the original studies included in this review, contributed to the drafting, and revised the manuscript.

## Conflict of Interest

The authors declare that the research was conducted in the absence of any commercial or financial relationships that could be construed as a potential conflict of interest.
